# Exploring the pathogenesis linking primary aldosteronism and obstructive sleep apnea via bioinformatic analysis

**DOI:** 10.1097/MD.0000000000039468

**Published:** 2024-09-06

**Authors:** Lanlan Zhao, Yuehua Dong, Ying Wei, Jie Li, Songyun Zhang

**Affiliations:** a Department of Endocrinology and Rare Diseases, The Second Hospital of Hebei Medical University, Shijiazhuang, China; b Department of Endocrinology, Baoding No. 1 Central Hospital, Baoding, China; c Hebei Key Laboratory of Rare Diseases, Shijiazhuang, China; d Porphyria Multi Disciplinary Team of the second Hospital of Hebei Medical University, Shijiazhuang, China.

**Keywords:** aldosterone-producing adenoma, bioinformatics, obstructive sleep apnea, primary aldosteronism, WGCNA

## Abstract

Primary aldosteronism (PA) and obstructive sleep apnea (OSA) are both considered independent risk factors for hypertension, which can lead to an increase in cardiovascular disease incidence and mortality. Clinical studies have found a bidirectional relationship between OSA and PA. However, the underlying mechanism between them is not yet clear. This study aims to investigate the shared genetic characteristics and potential molecular mechanisms of PA and OSA. We obtained microarray datasets of aldosterone-producing adenoma (APA) and OSA from the gene expression omnibus (GEO) database. Weighted gene co-expression network analysis (WGCNA) was used to select co-expression modules associated with APA and OSA, and common genes of the two diseases were obtained by intersection. Subsequently, hub genes for APA and OSA were identified through functional enrichment analysis, protein-protein interaction (PPI), datasets, and public database. Finally, we predicted the transcription factors (TFs) and mirRNAs of the hub genes. In total, 52 common genes were obtained by WGCNA. The Gene Ontology (GO) of common genes includes interleukin-1 response, cytokine activity, and chemokine receptor binding. Functional enrichment analysis highlighted the TNF, IL-17 signaling, and cytokine-cytokine receptor interactions related to APA and OSA. Through PPI, datasets, and public databases verification, we identified 5 hub genes between APA and OSA (IL6, ATF3, PTGS2, CCL2, and CXCL2). Our study identified shared 5 hub genes between APA and OSA (IL6, ATF3, PTGS2, CCL2, and CXCL2). Through bioinformatics analysis, we found that the 2 disorders showed relative similarity in terms of inflammation, stress, and impaired immune function. The identification of hub genes may offer potential biomarkers for the diagnosis and prognosis of PA and OSA.

## 1. Introduction

Primary aldosteronism (PA) is a common cause of secondary hypertension with a prevalence rate of approximately 5% to 10%. The characteristics of PA include excessive aldosterone secretion and inhibition of the renin-angiotensin system. Aldosterone-producing adenoma (APA) is a common subtype of PA, accounting for about 40% to 50% of PA cases.^[1]^ Compared with essential hypertension, the incidence of stroke and myocardial infarction in PA patients is significantly higher, so early diagnosis and active treatment are crucial.^[2]^ Obstructive sleep apnea (OSA) is a common sleep-related disorder. In recent years, with the rise in overweight and obese individuals, along with population aging, the occurrence of OSA has been escalating, becoming a pressing public health concern.^[3]^ OSA poses a significant threat to life safety and may result in accidental deaths and sudden fatalities during sleep.^[4]^ Furthermore, it exhibits a strong correlation with metabolic dysregulation, cardiovascular and nervous system impairments, as well as memory decline and cancer development.^[5]^ Multiple studies reveal a close link between OSA and PA. A cross-center, multi-ethnic study found that 67.6% of PA patients had OSA, with a significant correlation between plasma aldosterone and OSA severity.^[6]^ Nakamura et al discovered that 55% (39 of 71) of Japanese PA patients had OSA.^[7]^ Therefore, conducting OSA examinations and implementing management strategies for PA patients is imperative. A meta-analysis revealed that OSA affects nearly 46% of 2335 PA patients (95% CI = 39–54%). Notably, hypertensive patients with PA have a significantly higher OSA prevalence compared to those without PA (OR = 2.01, 95% CI = 1.37–2.95, *P* < .001). In addition, the study demonstrated that approximately 27% of 3498 OSA patients also had PA (95% CI = 24–29%). Notably, the prevalence of PA was significantly higher in OSA patients compared to non-OSA patients (OR = 2.03, 95% CI = 1.30–3.16, *P* = .002).^[8]^ Di Murro et al reported that approximately 34% of hypertensive patients with OSA also have PA.^[9]^ Increasing research indicates that there is a complex interaction between OSA and PA, and they are likely to share some pathological mechanisms.^[10,11]^ Despite numerous studies exploring the pathophysiological links between PA and OSA, the search for common genetic factors remains inadequate.

With the rapid advancements in bioinformatics, we can delve deeper into complex biological processes involving multiple genes. This study aims to identify crucial shared genes in the pathogenesis of APA and OSA, revealing their potential molecular mechanisms and offering new candidate genes for future diagnostic and therapeutic approaches.

## 2. Materials and methods

### 2.1. Microarray data and study design

**Figure 1.**
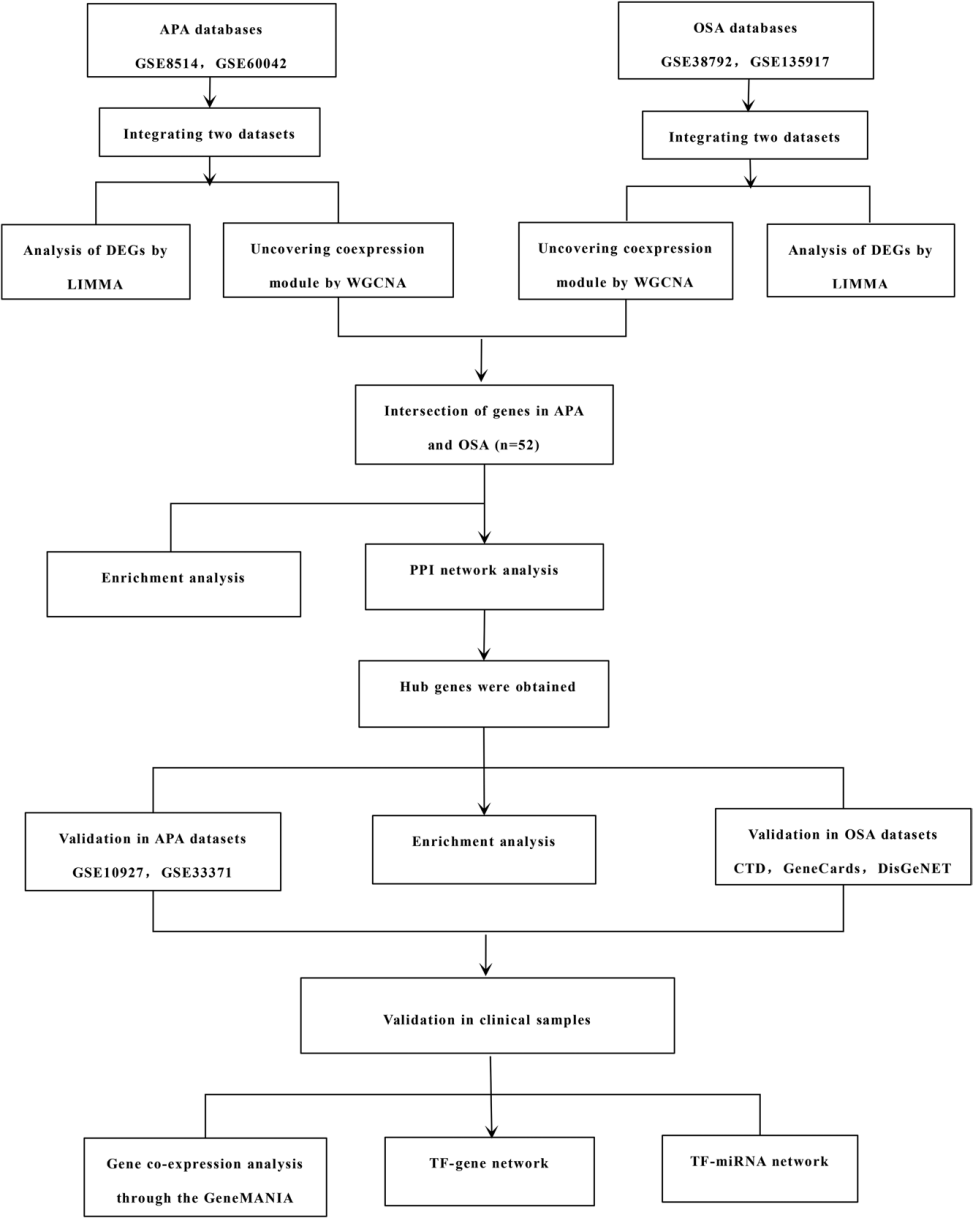
Study flowchart. APA = aldosterone-producing adenoma, DEGS = differentially expressed genes, OSA = obstructive sleep apnea, PPI = protein-protein interaction, TF = transcription factor, WGCNA = weighted gene co-expression network analysis.

We retrieved the expression profiling datasets for OSA and APA from the Gene Expression Omnibus using the keywords “OSA” and “APA,” respectively (https://www.ncbi.nlm.nih.gov/geo/). The study type was specified as “Expression profiling by array,” and the organisms were limited to “Homo sapiens.” The selected datasets include both case and healthy control samples, with no fewer than 12 samples (control samples + disease samples) in each dataset. As fat tissue plays a crucial role in the pathophysiology of OSA, we have selected a dataset related to fat tissue from OSA patients for further analysis (GSE135917, GSE38792). The datasets GSE135917 and GSE38792 each comprise adipose tissue samples from 10 patients with OSA and 8 healthy controls, matched based on criteria including gender, age, and BMI. APA primarily occur in the adrenal cortex. Therefore, conducting an in-depth analysis of this tissue can provide a better understanding of the pathogenesis of APA. The GSE8514 data set contains 10 APA adrenal tissues and 5 normal control adrenal tissues. The GSE60042 data set included 7 APA and 7 adjacent adrenal tissues (AGG). The study flowchart is demonstrated in Figure 1.

### 2.2. Data processing and differentially expressed genes (DEGs)

Firstly, the average expression levels of duplicated genes are computed. Next, the gene expression levels in the dataset are transformed using the Log2 function. Subsequently, the Bioconductor “limma” package (“normalizeBetweenArrays” function) is used for calibration and normalization. Further integration of the OSA datasets (GSE135917 and GSE38792) and APA datasets (GSE60042 and GSE8514) is performed, utilizing the “combat” algorithm from the “sva” package to eliminate batch effects between different reports and platforms.^[12]^ Probe sets without corresponding gene symbols are removed. Finally, genes with |log2 Fold change (FC)| > 0.585 and a *P* value < .05 are identified as DEGs.

### 2.3. Construction of weighted gene co-expression network analysis (WGCNA) and identification of key modules

WGCNA is a method to analyze the correlation between a module and a specific trait or phenotype, which is widely used.^[13]^ We utilized the “WGCNA” package in the R software to construct a co-expression network for all genes in OSA, APA samples, and normal control samples. Firstly, R language is used for hierarchical cluster analysis of the Hclust function, and outlier samples are excluded. Secondly, we choose a suitable *β* value as a soft threshold to construct a scale-free network. Then, the topological overlap matrix is established according to the formula. In this study, modules with high gene significance and *P* < .05 were considered highly correlated with clinical characteristics. Finally, through an online platform (http://bioinformatics.psb.ugent.be/webtools/Venn/), common genes were obtained using Venn diagrams from modular genes that were highly correlated with the clinical features of the two disorders.

### 2.4. Functional enrichment analysis

Gene Ontology (GO) includes biological process, molecular function (MF), and cell component (CC).^[14]^ The Kyoto Encyclopedia of Genes and Genomes (KEGG) is a database for understanding the advanced functions of biological systems.^[15]^ The functional enrichment analysis mainly used the R packages “clusterProfiler,” “enrichplot,” “complexHeatmap” and “ggplot2.” A *P* value < .05 was considered statistically significant.

### 2.5. Protein-protein interaction (PPI) establishment and hub genes identification

Further exploration of the interactions between these common genes could improve our understanding of how proteins function. PPI network was performed using STRING (http://string-db.org) for APA and OSA common genes, all parameters were set to default values.^[16]^ Analyzed and visualized PPI networks through Cytoscape (version 3.8.0). The key functional modules were analyzed by molecular complex detection (filter criteria: degree cut-of = 2; node score cut-of = 0.2; k-core = 2; max depth = 100). Then, the shared genes with more interactions are screened according to the Degree algorithm via the Cytohubba plug-in of Cytoscape. Subsequently, the R packages “clusterProfiler,” “enrichplot,” “complexHeatmap,” and “ggplot2” were applied to perform GO and KEGG analysis to hub genes.^[17]^

### 2.6. Validation of hub genes in datasets and public databases

We obtained 2 other APA public databases (GSE33371, GSE10927) to confirm the hub genes expression, and *P *< .05 was considered significant. Due to the lack of OSA data sets, we collected genes related to OSA through CTD (https://ctdbase.org/), GeneCards (https://www.genecards.org/), and DisGeNET (http://www.disgenet.org) databases. Next, the shared genes of the OSA databases, APA datasets, and 10 hub genes are identified through Venn diagrams. Finally, coexpression networks of hub genes were constructed using GeneMANIA (http://www.genemania.org).

### 2.7. Validation of hub genes related proteins in clinical peripheral blood samples

Yellow venous blood vessels containing coagulants were used to collect 5 mL of venous blood samples from 4 OSA patients, 4 PA patients, and 4 normal individuals matched in terms of gender, age, and BMI. The collected blood was allowed to stand at room temperature for 1 hour to naturally clot and serum precipitate. Subsequently, the samples were centrifuged at 3000 rpm for 20 minutes to separate the serum, which was then stored at −80°C for further processing. The concentrations of C-C motif chemokine ligand 2 (CCL2), C-X-C motif chemokine ligand 2 (CXCL2), and Interleukin-6 (IL-6) in the serum were determined using ELISA kits provided by ABclona, including the Human CCL2 ELISA kit, Human CXCL2 ELISA kit, and Human IL-6 ELISA kit. The concentrations of activating transcription factor 3 (ATF3) and prostaglandin-endoperoxide synthase 2 (PTGS2) in the serum were measured using Human ATF3 enzyme-linked immunosorbent assay kits and Human PTGS2 enzyme-linked immunosorbent assay kits provided by Shanghai Aimond Biotechnology Co., Ltd. This study adhered to the ethical standards established by the Ethics Committee of the Second Hospital of Hebei Medical University, and written informed consent was obtained from all participants before enrollment. All relevant guidelines and regulations were followed in this study.

### 2.8. Transcription factor (TF)-gene interactions and TF-miRNA coregulatory network

TFs are the key molecule of gene expression regulation. This study predicted the TFs of core genes through the TRRUST database (https://www.grnpedia.org/trrust/). An adjusted *P* value < .05 was significant. We construct the visualization of TF-gene networks via Cytoscape. TF-miRNA coregulated networks were obtained through NetworkAnalyst 3.0 (https://www.networkanalyst.ca/) and visualization via Cytoscape.

## 3. Result

### 3.1. DEGs in APA and OSA

**Figure 2.**
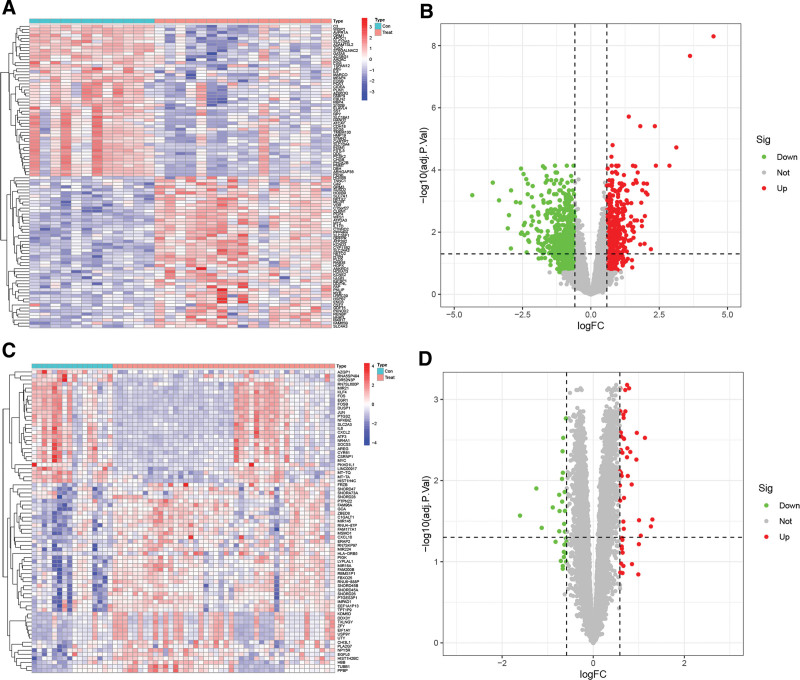
Heatmap and volcano plot for the DEGS identified from the APA dataset and OSA dataset. (A) Heatmap of the DEGS from the APA dataset. (B) Volcano plot of the DEGS from the APA dataset. (C) Heatmap of the DEGS from the OSA dataset. (D) Volcano plot of the DEGS form the OSA dataset. APA = aldosterone-producing adenoma, DEGS = differentially expressed genes, OSA = obstructive sleep apnea.

In total, 1207 DEGs were identified from the APA. The heatmap and volcano plot of DEGs were shown in Figure 2A and B. Similarly, we used the “Limma” package to identify 75 DEGs from the OSA merged data set and then mapped the heatmap and volcano plot (Fig. 2C and D).

### 3.2. Construction of WGCNA and identification of key module

**Figure 3.**
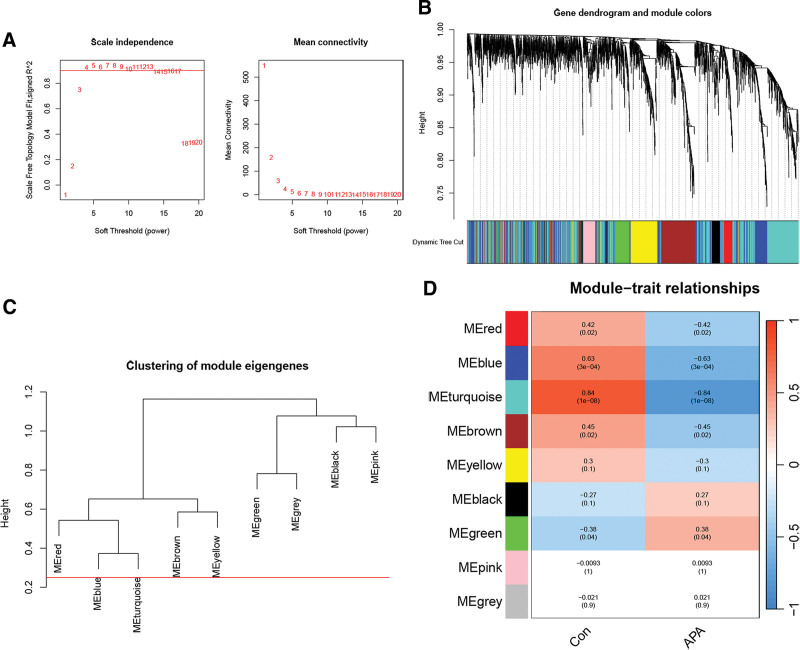
WGCNA construction in APA. (A) The left picture is used to determine the optimal soft threshold, and the right picture shows the network connectivity under different soft thresholds. (B) Gene dendrogram and module colors. (C) Dendrogram of characteristic genes of consensus modules obtained by WGCNA. (D) Eigengene adjacency heatmap of 8 modules. APA = aldosterone-producing adenoma, WGCNA = weighted gen co-expression network analysis.

WGCNA is used to identify disease-related modules in APA and OSA. In APA, after hierarchical clustering analysis and the elimination of batch effects, we chose*β* = 4 (scale-free *R*^2^ = 0.92) as the “soft” threshold (Fig. 3A). A total of eight gene modules, including black, blue, brown, green, gray, pink, red, turquoise, and yellow, were identified by hierarchical clustering and dynamic tree cutting algorithm (Fig. 3B–D). Each color represents a gene block. We identified the modules that were associated with APA, including the turquoise module (1180 genes), blue module (452 genes), red module (154 genes), and brown module (441 genes). Therefore, these modules were identified as key modules of APA for further analysis.

**Figure 4.**
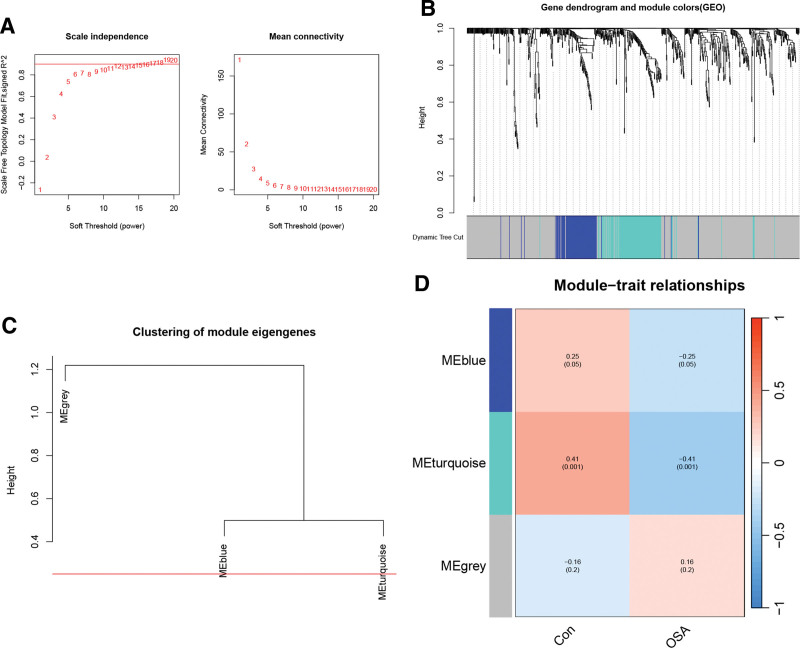
WGCNA construction in OSA. (A) The left picture is used to determine the optimal soft threshold, and the right picture shows the network connectivity under different soft thresholds. (B) Gene dendrogram and module colors. (C) Dendrogram of characteristic genes of consensus modules obtained by WGCNA. (D) Eigengene adjacency heatmap of 2 modules. OSA = obstructive sleep apnea, WGCNA = weighted gen co-expression network analysis.

**Figure 5.**
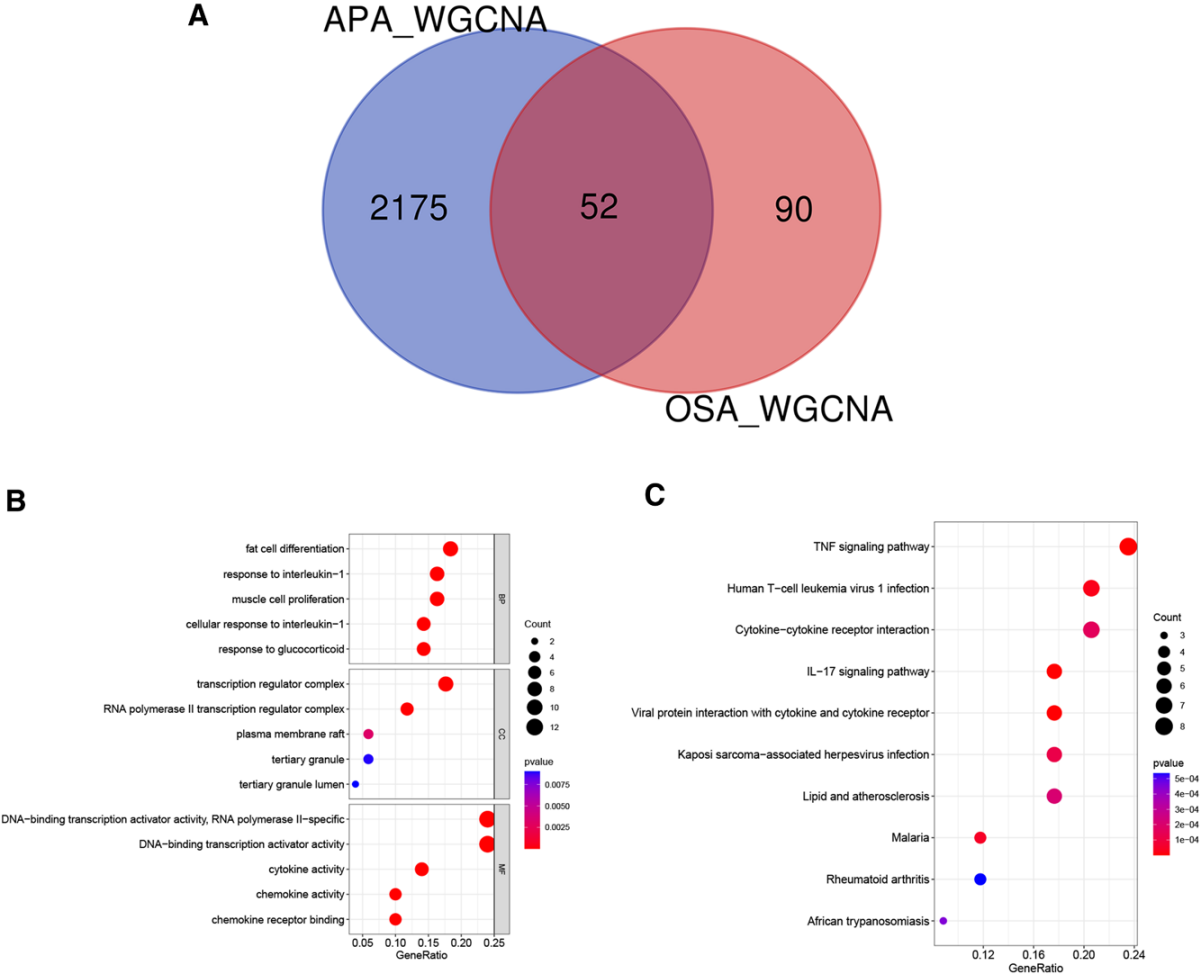
Venn diagram and Enrichment analysis. (A) The Venn diagram shows that 52 common genes were identified by WGCNA from the intersection of two diseases. (B) GO analysis of the common genes, including BP, CC, and MF respectively. (C) KEGG pathway analysis of the common genes. BP = biological process, CC = cell component, GO = gene ontology, KEGG = Kyoto Encyclopedia of Genes and Genomes, MF = molecular function, WGCNA = weighted gen co-expression network analysis.

In OSA, we chose *β* = 11 (scale-free *R*^2^ = 0.84) as the “soft” threshold (Fig. 4A). Two gene modules were identified by WGCNA network construction and average linkage hierarchy clustering. Detailed hierarchical clustering information is shown in Figures 4B–D. The correlation analysis showed that the turquoise module (142 genes) was the key module of OSA. We use the “WGCNA” package in R software to execute WGCNA. Subsequently, we obtained 52 common genes from APA and OSA-related gene modules (4 APA gene modules, 1 OSA gene module) in WGCNA through a Venn diagram (Fig. 5A).

### 3.3. Functional enrichment analysis

To understand the biological functions of common genes, we conducted enrichment analysis, including GO and KEGG. The biological process of common genes is mainly enriched in adipocyte differentiation, interleukin-1 response, and glucocorticoid response. In addition, cytokine activity, chemokine receptor binding, and other CC may be related to APA-OSA common genes. These common genes are related to MF such as RNA polymerase Ⅱ transcription, cytokine, and chemokine activity. The KEGG of common genes is mainly involved in the tumor necrosis factor (TNF), IL-17 signaling, and cytokine-cytokine receptor interaction (Fig. 5B and C).

### 3.4. PPI establishment and hub genes identification

**Figure 6.**
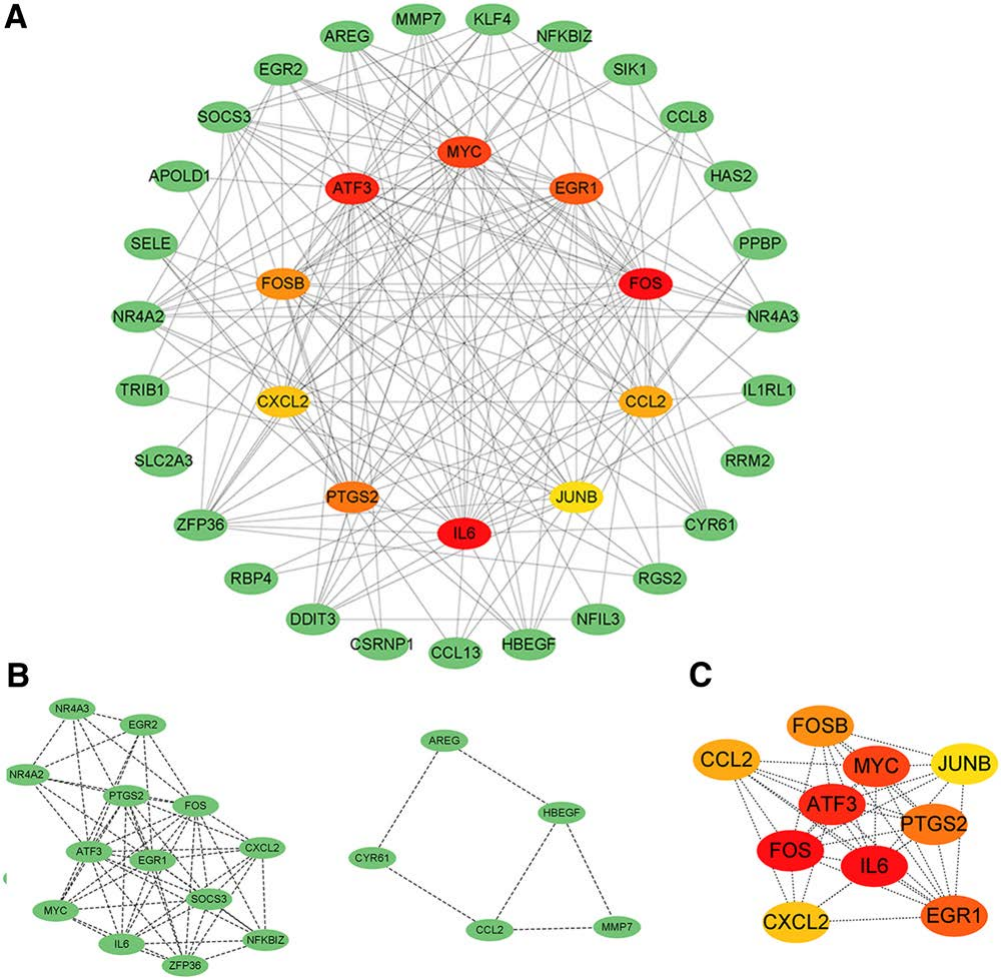
PPI network analysis. (A) PP1 network of common genes among APA and OSA. In the figure, the nodes of the central circle represent the hub genes. (B) Two cluster modules extracted by MCODE. (C) The 10 hub genes screened by degree through the cytohubba. MCODE = molecular complex detection, PPI = protein-protein interaction.

We constructed a PPI network of the 52 common genes using the STRING database. The PPI network, which contained 44 nodes and 186 edges, was constructed and analyzed using Cytoscape (Fig. 6A). We have acquired two related gene modules via Cytoscape’s molecular complex detection. Module 1 consisted of 13 nodes and 56 edges, with a clustering score of 9.33. Module 2 contains 5 nodes and 6 edges, with a clustering score of 3 (Fig. 6B). CytoHubba was applied to identify 10 genes from highest to lowest according to node connectivity (Degree) as the hub genes for the two diseases: IL6, FOS, ATF3, MYC, EGR1, PTGS2, FOSB, CCL2, CXCL2, and JUNB (Fig. 6C).

**Figure 7.**
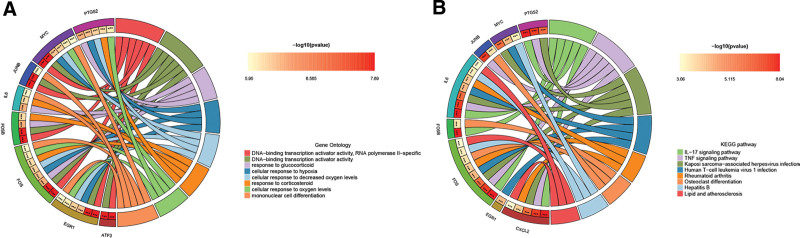
Enrichment analysis of hub genes. (A) GO analysis of hub genes. (B) KEGG pathway analysis of the hub genes. GO = gene ontology, KEGG = Kyoto Encyclopedia of Genes and Genomes.

Subsequently, GO analysis showed that these hub genes were associated with DNA-binding transcriptional activator activity, RNA polymerase ll-specific, cell response to hypoxia, glucocorticoid response, etc. KEGG analysis primarily enriched in Interleukin-17 (IL-17) pathway, tumor necrosis factor (TNF) signaling, lipid and atherosclerosis (Fig. 7A and B).

### 3.5. Validation of hub genes in public databases and datasets

**Figure 8.**
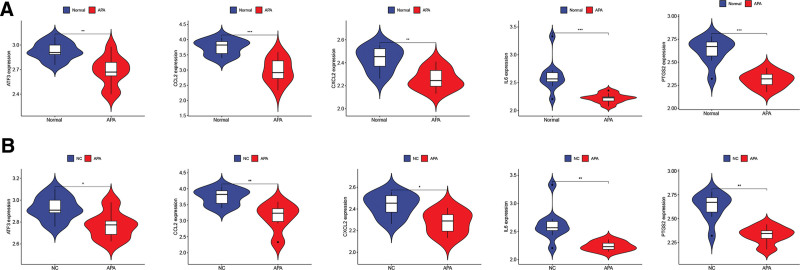
The validation of hub genes in APA data sets. (A) Comparison between 2 sets of data in GSE10927. (B) Comparison between two sets of data in GSE33371. APA = aldosterone-producing adenoma.

**Figure 9.**
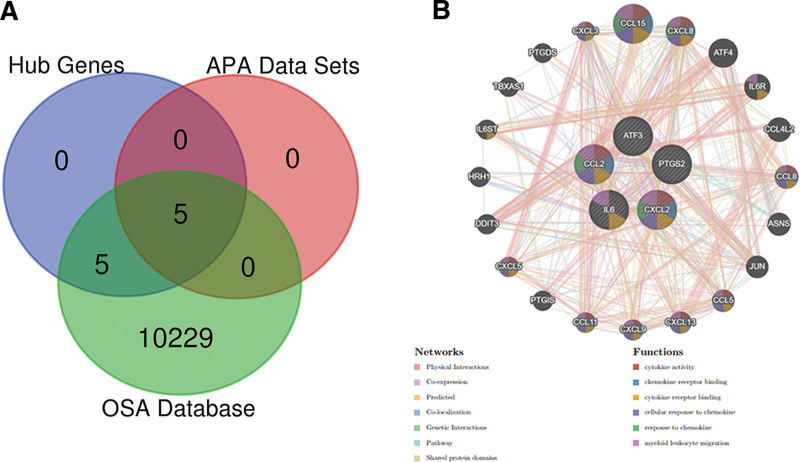
Venn diagram and co-expression network of hub genes. (A) Venn diagram of hub genes, APA data sets and OSA database (CTD, GeneCards, DisGeNET). (B) Core genes and their co-expression genes were analyzed via GeneMANIA. APA = aldosterone-producing adenoma, OSA = obstructive sleep apnea.

We verified these hub genes in two other APA public databases (GSE33371, GSE10927). The results showed that the expressions of IL6, ATF3, PTGS2, CCL2, and CXCL2 were different between APA and the control groups (Fig. 8A and B). We obtained 10,239 OSA-related genes by searching, de-replicating, and merging from CTD, GeneCards, and DisGeNET databases. We then identified 5 hub genes (IL6, ATF3, PTGS2, CCL2, and CXCL2) from the hub genes, APA datasets, and OSA databases through the Venn diagram (Fig. 9A). Based on the GeneMANIA database, five hub genes display a complicated PPI network, including 8.01% co-expression, 77.64% physical interaction, 3.63% co-localization, 5.37% prediction, and 1.88% pathways (Fig. 9B).

### 3.6. Validation of hub genes related proteins in clinical peripheral blood samples

**Figure 10.**
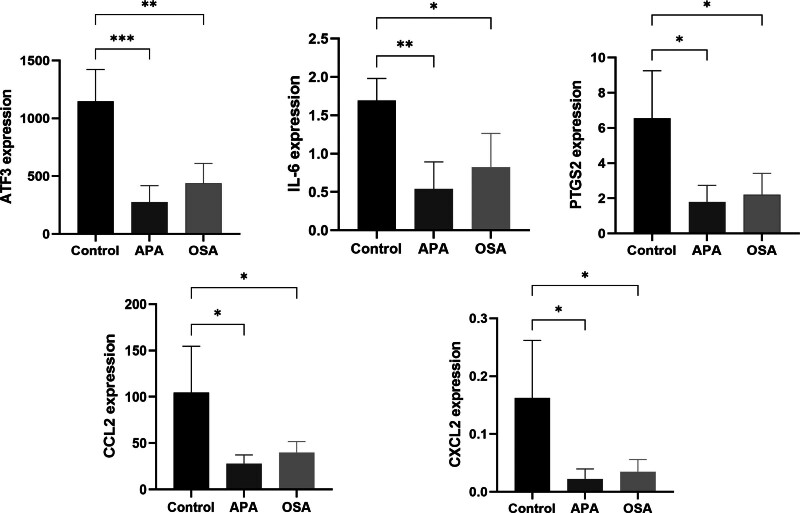
Validation of hub genes related proteins in clinical peripheral blood samples. *P < .05; **P < .01; ***P < .001.

To further validate the expression results obtained from bioinformatics studies, ELISA assays were conducted to confirm the levels of serum IL-6, ATF3, PTGS2, CCL2, and CXCL2 in OSA patients, PA patients, and control individuals. The results revealed that compared to the control group, the serum concentrations of IL-6, ATF3, PTGS2, CCL2, and CXCL2 were significantly elevated in both OSA and APA patients (Fig. 10).

### 3.7. TF-gene interactions and TF-miRNA coregulatory network

**Figure 11.**
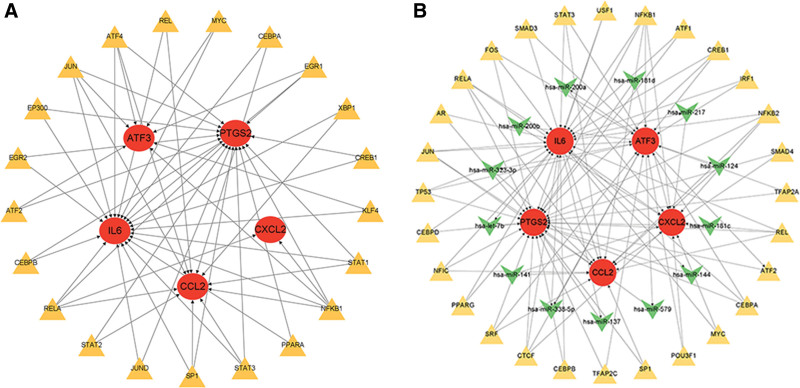
Network of TF-gene and TF-miRNA. (A) The network presents the TF-gene coregulatory network. (B) The network presents the TF-miRNA coregulatory network. The highlighted red color nodes represent the core genes.

We identified 21 TFs that may regulate these 5 hub genes through the TRRUST database. The TF-gene network contains 26 nodes and 55 edges. The degree value of transcription factor nuclear factor-k-gene binding 1 in the TF - gene interaction network is 5. In the TF-gene interaction network, PTGS2 is regulated by 16TFs, and IL6 is regulated by 19TFs (Fig. 11A). In the TF-miRNA network, 13 miRNAs interact with 5 core genes. In the TF-miRNA network, PTGS2, CXCL2, and ATF3 all interact with 6 miRNAs (Fig. 11B).

## 4. Discussion

Both PA and OSA are prevalent causes of refractory hypertension, often resulting in unfavorable cardiovascular outcomes.^[18,19]^ The coexistence of excessive aldosterone secretion and OSA is particularly prevalent among patients with resistant hypertension, obesity, and metabolic syndrome. Recent studies have suggested a potential association between patients with refractory hypertension and a high incidence of OSA, indicating a possible bidirectional relationship between the two conditions.^[20]^ On one hand, excessive aldosterone secretion leads to water and sodium retention, causing fluid overload in the body, not only increasing fluid load but also worsening airflow obstruction, which plays a crucial role in the development of OSA. Conversely, intermittent hypoxia can further activate the renin-angiotensin-aldosterone syste (RAAS), leading to hyperaldosteronism.^[21]^ Moreover, excess fat tissue and its secretions in obese individuals may contribute to the occurrence of sleep apnea and aldosterone overproduction. On the other hand, PA patients can effectively reduce the occurrence of OSA and significantly improve oxygen saturation levels through mineralocorticoid receptor blockers or adrenal gland resection.^[22]^ Additionally, continuous positive airway pressure (CPAP) therapy has been proven to significantly reduce the activity of the RAAS in OSA patients.^[23]^ Although the Endocrine Society guidelines in 2016 explicitly recommended screening for PA in patients with comorbid hypertension and OSA, in practical application, we still grapple with the dilemma of limited diagnostic methods and the need for improved accuracy. Therefore, the search for novel and more precise diagnostic biomarkers is crucial. Numerous renowned scholars have conducted in-depth bioinformatics analyses on the potential genes and biomarkers for OSA^[24,25]^ and PA in past studies,^[26,27]^ accumulating a wealth of knowledge in this field. However, genetic studies on shared genes and potential mechanisms between PA and OSA remain relatively scarce and urgently require further exploration. In order to further elucidate the potential link between OSA and PA, we utilized bioinformatics analysis techniques to explore their possible shared genes and underlying mechanisms, with the aim of identifying potential diagnostic and therapeutic targets, and providing more precise and effective strategies for the prevention and treatment of this disease.

For the first time, we employed WGCNA to analyze APA and OSA datasets, aiming to identify DEGs. Through meticulous bioinformatics analysis, we discovered a total of 10 hub genes that are common to both APA and OSA patients. Subsequently, through datasets and clinical samples, it was found that among all hub genes, IL6, ATF3, PTGS2, CCL2, and CXCL2 were more closely related to OSA and APA. Moreover, through functional enrichment analysis of these hub genes, we observed that the molecular functions, biological processes, and cell composition related to APA and OSA genes are similar. Notably, these genes are primarily associated with fat cell differentiation, DNA-binding transcription activator activity, chemokine activity, and response to glucocorticoid. Finally, our pathway analysis uncovered several shared inflammatory and immune signaling pathways between APA and OSA, encompassing cytokine-cytokine receptor interactions, IL-17 and TNF signaling pathway.

Obesity and chronic intermittent hypoxia (CIH) are key features of OSA.^[28]^ In OSA patients, CIH increases relative oxygen production, disrupting the balance between oxidative and antioxidative processes, leading to increased oxidative stress. During CIH, blood redistribution occurs, stimulating the sympathetic nervous system and decreasing extracellular fluid volume. This physiological response triggers renin secretion from juxtaglomerular cells, activating the RAAS. ATF3, a member of the CREB/ATF family, plays a crucial role in regulating processes such as hypoxia, inflammation, immune-related diseases, and metabolism.^[29,30]^ It is a well-known stress response mediator that is quickly activated in response to stressors such as hypoxia, inflammation, and exposure to lipopolysaccharides. Our research has identified ATF3 as a gene commonly shared by OSA and APA. Given its multifaceted functions, we hypothesize that the relationship between OSA and APA may be closely tied to hypoxia, inflammation, and immunity. However, there is a lack of research on the role of ATF3 in OSA and APA patients. Interestingly, contrary to previous findings by Wu et al, our study found decreased ATF3 expression levels in adipose tissue in OSA patients.^[31]^ Ku et al^[32]^ research revealed that ATF3 gene-deficient mice are more prone to obesity and insulin resistance under a high-fat diet than wild-type mice. Notably, ATF3 inducers inhibit adipogenesis and activate related metabolic pathways, underscoring the critical role of ATF3 in fat metabolism. Felizola et al^[33]^ discovered that angiotensin II stimulates the expression of ATF3 miRNA levels, suggesting a potential role of ATF3 in aldosterone synthesis in the adrenal cortex. In addition, a complex association between the ATF3 gene and tumor cells has been noted, however its exact involvement in APA requires further investigation. Based on the above evidence, we think that the reduced expression of ATF3 observed in patients with OSA and APA in this study may contribute to the exacerbation of inflammation and immune response, primarily through the enhancement of obesity and adipogenesis, along with the attenuation of its anti-inflammatory capabilities.

Abnormal aldosterone secretion in patients with APA can stimulate the immune system, leading to increased activity of macrophages and lymphocytes. This activation promotes the release of inflammatory mediators, initiating an inflammatory response. Furthermore, the primary pathophysiological mechanism of OSA is strongly associated with systemic inflammation. This study revealed that CXCL2, IL-6, CCL2, and PTGS2 are among the genes that are commonly found in both OSA and APA. Previous studies have revealed that patients with OSA exhibit significantly elevated serum expression levels of chemokines including IL-6, PTGS2, and monocyte chemokine protein-1 (MCP-1).^[34–36]^ Analogously, individuals afflicted with APA also demonstrate increased concentrations of IL-6, PTGS2, and MCP-1 in their serum.^[37,38]^ MCP-1, alternatively known as CCL2, is activated by inflammatory cells like monocytes/macrophages and other cytokines at the site of inflammation, promoting migration and infiltration.^[39,40]^ Macrophage inflammatory protein-2, known as CXCL2, is primarily secreted by activated monocytes and neutrophils, playing a pivotal role in immune regulation and inflammatory processes.^[41]^ cyclooxygenase-2, encoded by the PTGS2 gene, plays a crucial role in inflammatory responses. It is worth noting that existing literature reports a significant decrease in IL-6 and MCP-1 levels in OSA patients after receiving CPAP treatment.^[42,43]^ Additionally, IL-6 inhibitors have been proven effective in improving aldosterone-induced cardiac fibrosis.^[38]^ Our findings revealed a significant down-regulation in the expression levels of inflammation-related genes CXCL2, IL-6, CCL2, and PTGS2 in OSA and APA samples compared to the control group, contradicting previous research. In the datasets GSE135917 and GSE38792, the BMI of both OSA patients and the control group are matched and classified as obese, which may have influenced the expression levels of inflammatory factors in the control group. Additionally, the control samples in the GSE60042 dataset are all derived from adjacent adrenal glands of APA patients, while the adrenal samples in the control group of the GSE8514 dataset come from patients who underwent adrenalectomy due to kidney cancer. This suggests that there are similarities in hormonal exposure between the control and disease groups, which could potentially influence the results.

This study found that the common genes identified in both APA and OSA exhibited significant enrichment in inflammation-related signaling pathways, particularly IL-17 and TNF. IL-17, secreted by T helper cell 17 (Th17), plays a crucial role in initiating and mediating proinflammatory responses.^[44]^ When IL-17 synergizes with other cytokines, it can jointly prompt immune cells to produce more proinflammatory mediators, thereby enhancing the immune-inflammatory response. Consistent with Wu et al ‘s study, the genes identified in this study are significantly enriched in IL-17 and TNF signaling pathways in OSA patients.^[31]^ Liu et al discovered that in patients with OSA, the proportion of Th17 cells, the ratio of Th17/Treg, and serum IL-17 levels were positively correlated with the apnea-hypopnea index and negatively correlated with the lowest oxygen saturation.^[45]^ Furthermore, research conducted by Shen et al suggests that CPAP therapy may potentially alleviate the imbalance between Th17 and Treg cells in OSA patients and reduce the secretion of proinflammatory cytokines.^[46]^ Aldosterone plays a promoting role in inflammatory states, characterized by the infiltration of immune cells into the vasculature, reactive oxidative stress, and the production of proinflammatory cytokines. Research indicates that mineralocorticoid-induced hypertension seems to be closely associated with the involvement of cells of the adaptive immune system, particularly T cells.^[47]^ Furthermore, the research conducted by Imiela et al has further clarified that aldosterone enhances the polarization of Th17 cells, leading to a disruption in the balance between Th17 and Treg cells.^[48]^ This imbalance may potentially have significant effects on aldosterone-induced cardiovascular and renal fibrosis. TNF-α is an important proinflammatory cytokine primarily secreted by macrophages, crucial for maintaining host defense mechanisms, and intricately linked to the pathogenesis of various diseases. Research has demonstrated the crucial role of TNF-α in sleep regulation, particularly its close association with phenomena such as daytime sleepiness, nocturnal sleep disruption, and hypoxia.^[49]^ In patients with OSA, serum and plasma levels of IL-6 and TNF-α are significantly elevated, and these levels positively correlate with disease severity, age, and BMI. Previous studies have indicated that patients with primary hypertension have significantly higher levels of TNF-α compared to those with PA.^[50]^ Additionally, Zhang et al^[51]^ discovered that aldosterone can trigger the production of proinflammatory cytokines, including TNF-α, IL-1β, and MCP-1.

Despite its limitations, this study provides a new perspective for exploring the pathogenesis of PA and OSA. Firstly, as the study relies on a retrospective analysis of public databases, there may be limitations in terms of data integrity and accuracy. Therefore, future prospective studies will require more rigorous designs and larger sample sizes to validate these findings. Secondly, while genes common to both OSA and APA have been identified, the biological function of these genes in APA remains unclear. Therefore, our future endeavors will be aimed at elucidating the biological role of these genes in both diseases.

## 5. Conclusion

In this study, we identified five core genes (IL6, ATF3, PTGS2, CCL2, and CXCL2) that were commonly shared between APA and OSA. We speculate that inflammation, stress, and immune function damage may serve as common pathogenic mechanisms underlying APA and OSA. This discovery carries significant implications for deepening our understanding of the pathogenesis of APA and OSA, as well as developing more effective diagnosis and treatment methods.

## Acknowledgments

We thank the subjects who donated the samples, as well as the researchers who created and shared a large amount of data.

## Author contributions

**Conceptualization:** Lanlan Zhao.

**Data curation:** Ying Wei.

**Investigation:** Ying Wei.

**Methodology:** Yuehua Dong.

**Resources:** Yuehua Dong.

**Software:** Lanlan Zhao.

**Supervision:** Jie Li, Songyun Zhang.

**Validation:** Lanlan Zhao, Yuehua Dong.

**Visualization:** Jie Li.

**Writing – original draft:** Lanlan Zhao.

**Writing – review & editing:** Lanlan Zhao.
